# Functionalized Derivatives of Benzo-Crown Ethers. Part 4. Antifungal Macrocyclic Supramolecular Complexes of Transition Metal Ions Acting as Lanosterol-14-α-Demethylase Ihibitors

**DOI:** 10.1155/MBD.1999.101

**Published:** 1999

**Authors:** Mihai Barboiu, Claudiu T. Supuran, Andrea Scozzafava, Cornelia Guran, Paula Diaconescu, Mihaela Bojin, Vlad Iluc, Louis Cot

**Affiliations:** 1 Universitatea Politehnica Bucuresti Dept. of Analytical Chemistry 1, Polizu Bucuresti R-78126 Romania; 2 Laboratoire des Matériaux et procédés Membranaires Ecole Nationale Supérieure de Chimie Montpelier 8 rue de I'Ecole Normale Montpellier,Cedex 5 F-34296 France; 3 Università degli Studi Dipartamento di Chimica Laboratoiro di Chimica Inorganica e Bioinorganica Via Gino Capponi 7 Firenze I-50121 Italy; 4 Universitatea Politehnica Bucuresti Dept of lnorganic Chemistry 1,Polizu Bucuresti R-78126 Romania

## Abstract

Poly- and mononuclear metal complexes of 2,3,11,12-bis[4-(10-aminodecylcarbonyl)]benzo-18- crown-6 (L) and Cu(II); Ni(II); Co(II) and Cr(III) have been synthesized and characterized by standard physico-chemical procedures. In the newly prepared complexes the crown moiety oxygen atoms of the macrocyclic host did not generally interact with metal ions, whereas the two amino groups of the ligand always did. Several of the newly synthesized compounds act as effective antifungal agents against *Aspergillus* and *Candida spp.*, some of them showing activities comparable to ketoconazole, with minimum inhibitory concentrations in the range of 0.3−0.5 μg/mL. The mechanism of antifungal action of these coordination compounds is probably connected to an inhibition of lanosterol-14-α-demethylase, a metallo-enzyme playing a key role in sterol biosynthesis in fungi, bacteria and eukariotes.


### FUNCTIONALIZED DERIVATIVES OF BENZO-CROWN ETHERS. PART 4. ANTIFUNGAL MACROCYCLIC SUPRAMOLECULAR COMPLEXES OF TRANSITION METAL IONS ACTING AS LANOSTEROL-14-α-DEMETHYLASE INHIBITORS

Mihai Barboiu[1,2], Claudiu T. Supuran*[3], Andrea Scozzafava[3], Cornelia Guran[4], Paula Diaconescu[4], Mihaela Bojin[4], Vlad Iluc[4], and Louis Cot[2]

[1] Universitatea Politehnica Bucuresti, Dept. of Analytical Chemistry, 1, Polizu, R-78126, Bucuresti, Romania

[2] Laboratoire des Matériaux et Procédés Membranaires, Ecole Nationale Supérieure de Chimie Montpellier 8, rue de l'Ecole Normale, F-34296 Montpellier, Cedex 5, France

[3] Università degli Studi, Dipartamento di Chimica, Laboratorio di Chimica Inorganica e Bioinorganica, Via Gino Capponi 7, I-50121, Firenze, Italia

[4] Universitatea Politehnica Bucuresti, Dept. of Inorganic Chemistry, 1, Polizu, R-78126, Bucuresti, Romania

**Abstract**: Poly- and mononuclear metal complexes of 2,3,11,12-bis[4-(10-aminodecylcarbonyl)]benzo-18-crown-6 (L) and Cu(II); Ni(II); Co(II) and Cr(III) have been synthesized and characterized by standard physico-chemical procedures. In the newly prepared complexes the crown moiety oxygen atoms of the macrocyclic host did not generally interact with metal ions, whereas the two amino groups of the ligand always did. Several of the newly synthesized compounds act as effective antifungal agents against *Aspergillus* and *Candida spp.*, some of them showing activities comparable to ketoconazole, with minimum inhibitory concentrations in the range of 0.3 – 0.5 μg/mL. The mechanism of antifungal action of these coordination compounds is probably connected to an inhibition of lanosterol-14-α-demethylase, a metallo-enzyme playing a key role in sterol biosynthesis in fungi, bacteria and eukariotes.

### Introduction

Since the discovery of the crown-ether ligands in the late 1960s, these types of molecules have been recognized as specific complexing agents for alkaline and alkaline-earth metal ions [1][2] as well as for organic ammonium derivatives such as amines, amino acids, and related compounds (obviously in protonated state).[3]

Two type of complementarities between the metal ion and the crown ether can be distinguished. Firstly, when a metal ion directly fits into the cavity, interacting equally with all donor atoms present, spherical molecular recognition has been evidenced, leading thus to stable supramolecular complexes.[4] When the cavity size and the radius of the metal ions are not compatible with each other, out of cavity coordination or fractional coordination by other available sites has been evidenced in the isolated supramolecular complexes.[5][6] The latter case is observed for many transition metal ions: because of their small size (non-compatible with the relatively large crown cavity such as that of 18-crown-6) or their preferences for lower coordination numbers, they generally do not fit into such a macrocyclic cavity. For this reason only a small number of supramolecular complexes of transition metal ions with crown-ethers have been reported up to now.[5][6] Generally in such complexes, the hydrated metal ions form hydrogen bonded networks with heteroatoms present in the crown ether moiety, giving polymeric one- or two-dimensional derivatives.[5][6] In such cases, crown-ether molecules are hydrogen bonded through the metal bound water molecules.[6]

We describe here another approach for obtaining some novel complexes of transition metal ions with a functionalized macrocyclic crown-ether. According to this approach, the coordination sphere of the metal ions is completed by secondary coordination sites, chemically grafted on the macrocyclic cavity, which then participate in the molecular recognition processes together with the macrocycle. In this way the metal ion - macrocyclic receptor interactions become more intense, leading to new specific supramolecular arrays.

We have previously reported some ditopic macrocyclic derivatives containing different moieties in their second coordination sphere (such as amino, ammonium, pyrilium, pyridinium or *L*-amino acid groups). [7][8][9][10][11][12] Their complexation properties evidenced new multiple molecular recognition processes of the metal ions or of certain amino acids, due to the combination of different types of non-covalent interactions. [9][10] A selective membrane transport of $Ag^{+}/Cu^{++}$ by using functionalized dibenzo-18-crown-6 derivatives chemically grafted in a solid heteropolysiloxane matrix was also evidenced as being due to a synergetic supramolecular effect. [10][11]

In this paper we report the preparation of new supramolecular complexes of 2,3,11,12-bis[4-(10-aminodecylcarbonyl)]benzo-18-crown-6, **1** (L), previously reported by us [8][9][10][11][12] and various metal salts of the type $MCl_x$ y $H_2O$ (M=Cu(II), y=2; Ni(II),, y=6; Co(II), y=3; Cr(III), y=6). The new complexes were characterized by analytical and spectroscopic methods that allowed us to propose their structure, since good crystals for X-ray diffraction experiments could not be obtained.

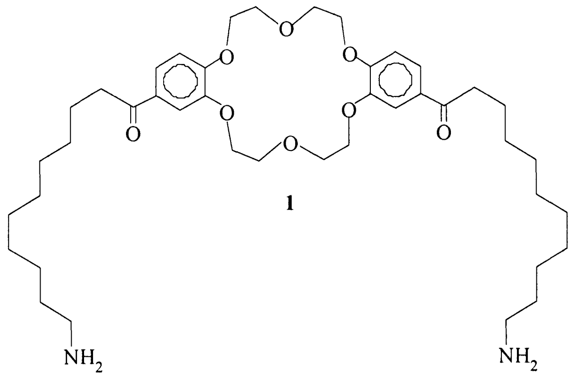

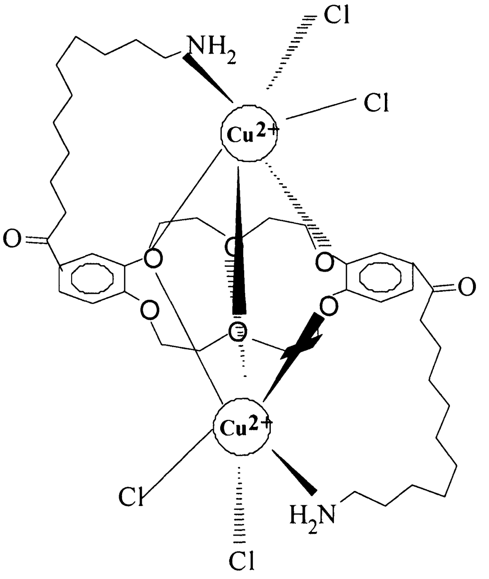

**2**

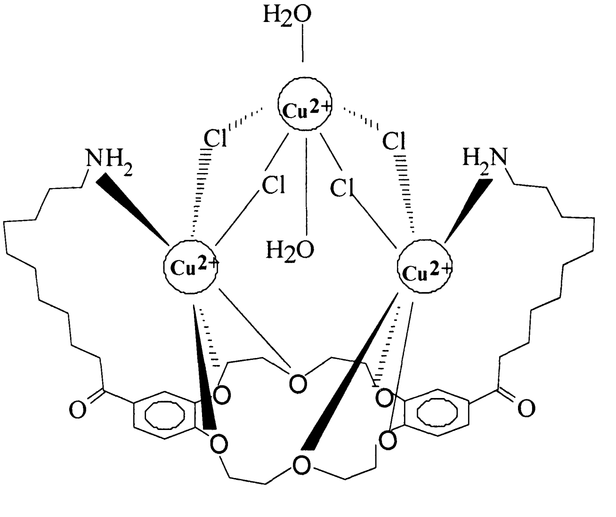

**3**

**Results and Discussion**

Treatment of bis-amine **1** with different hydrated metal salts ($MCl_x$ y $H_2O$: M=Cu(II), y=2; Ni(II), y=6; Co(II), y=3; Cr(III), y=6) led to a new series of supramolecular complexes of transition metal ions **2-9** (see Experimental).

The new supramolecular complexes **2-9** reported here were characterized by elemental analysis, IR and UV-VIS spectroscopy.

Elemental analysis data for compounds **2-9** were within ±0.5% of theoretical values calculated for the proposed formulae, evidencing different stoichiometries of combinations between the macrocyclic receptor **1** and the transition metal salts.

Table1: Selected IR bands of ligand **1** and its transition metal complexes **2-9**.

| Compound | $\nu_{C\text{-}O\text{-}Csim}$ [$cm^{-1}$] | $\nu_{C\text{-}O\text{-}Car}$ [$cm^{-1}$] | $\nu_{C\text{-}O\text{-}Cas}$ [$cm^{-1}$] | $\nu_{H2O}$ [$cm^{-1}$] |
|---|---|---|---|---|
| **1** | 1059 | 1136 | 1261 | - |
| **2** | 1055 | 1133 | 1264 | - |
| **3** | 1056 | 1131 | 1264 | 3448, 3620 |
| **4** | 1056 | 1131, 1148 | 1264 | 3405 |
| **5** | 1056 | 1131, 1148 | 1264 | 3368 |
| **6** | 1056 | 1132, 1148 | 1266 | 3437 |
| **7** | 1055 | 1125, 1149 | 1263 | 3554 |
| **8** | 1056 | 1129, 1148 | 1275 | 3458 |
| **9** | 1056 | 1130, 1148 | 1264 | 3445 |

In its IR spectrum, the diamine **1** exhibits two weak absorption bands at 3369 and 3438 $cm^{-1}$ attributable to the free (unassociated amino), antisymmetrical and symmetrical N-H vibrations, respectively, and at 1423-1456 $cm^{-1}$ , characteristic for the $\nu_{C\text{-}N}$ vibration. The shift with 100-150 $cm^{-1}$ towards lower wave numbers of the $-NH_2$ vibrations in the IR spectra of all the complexes reported here is indicative for metal coordination through the nitrogen atom(s). The $\nu_{C\text{-}N}$ vibration also undergoes a downfield shift with 5-10 $cm^{-1}$ in the new complexes as compared to the same vibration in the free ligand. Changes of the ether vibrations are also observed (table 1) on going from bis-amine **1** to its metal complexes, both for the symmetrical ($\nu_{C\text{-}O\text{-}C\ sim}$) as well as the antisymmetrical ($\nu_{C\text{-}O\text{-}C\ as}$ ; $\nu_{C\text{-}O\text{-}C\ Ar}$) aromatic ether stretchings, probably due to the participation of ether-oxygen atoms in the coordination process, or due to hydrogen bonding with water molecules.[13]

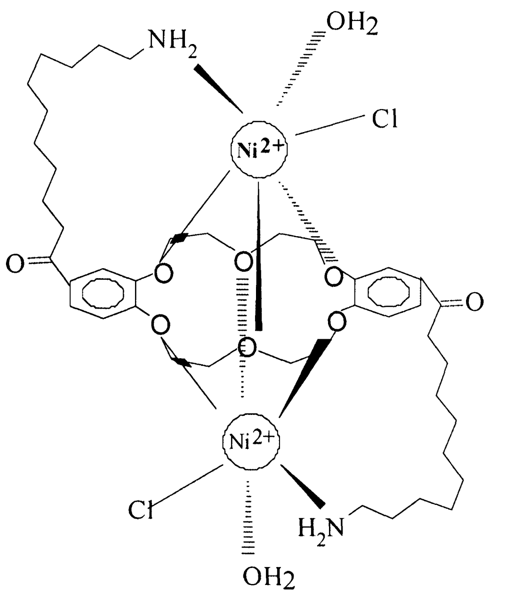

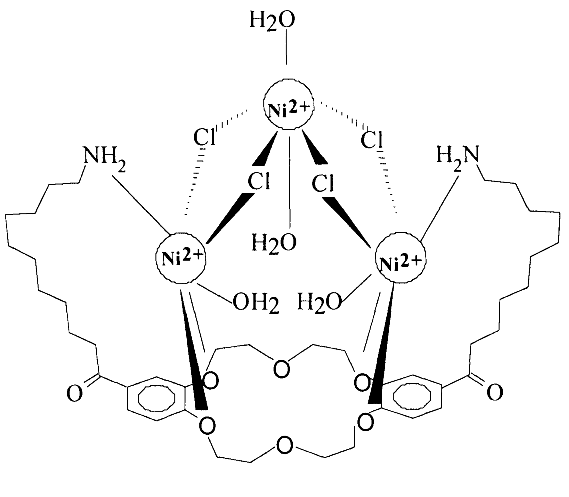

**4** **5**

Stereochemical information for the new complexes **2-9** was obtained from the absorption electronic spectra of these compounds. Thus, in the electronic spectra of the Cu(II) derivatives $\mathbf{Cu_2Cl_4L}$ **2** and $\mathbf{Cu_3Cl_4L(H_2O)_2}$ **3** a large band located in the range 10-18 x $10^3$ $cm^{-1}$ has been evidenced. By deconvoluting it using the Gauss method, [14] the following transitions bands were observed: at 11.35 x $10^3$, 14.37 x $10^3$, 15.7 x $10^3$ $cm^{-1}$ for complex **2** and 11.89 x $10^3$, 14.59 x $10^3$, 14.63 x $10^3$ $cm^{-1}$ for complex **3**, respectively, according to the $d_{z^2} \rightarrow d_{x^2-y^2}$, $d_{xy} \rightarrow d_{x^2-y^2}$ and $d_{xz}$, $d_{yz} \rightarrow d_{x^2-y^2}$ transitions. (Table 2). A second charge transfer band located at 18-25 x $10^3$ $cm^{-1}$ has been evidenced, which overlaps with some characteristic bands of the free ligand. [14] These spectra are characteristic for copper(II) ions in a (pseudo)-octahedral configuration and are consistent with a distorted $O_h$ geometry, the chromophores being of the $CuNO_3Cl_2$ type for complex **2** and the $CuNO_3Cl_2$ and $CuCl_4O_2$ types for complex **3**.

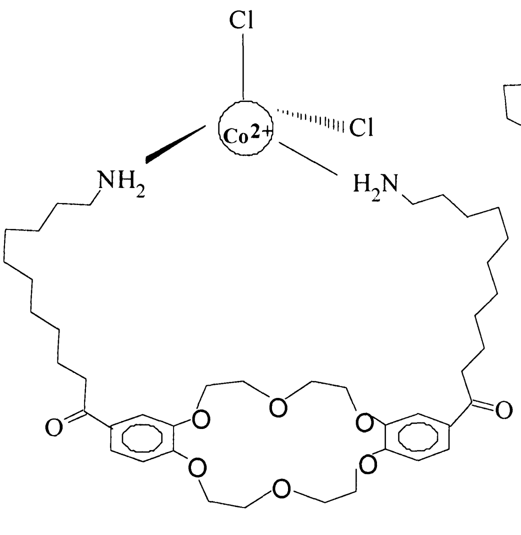

**6**

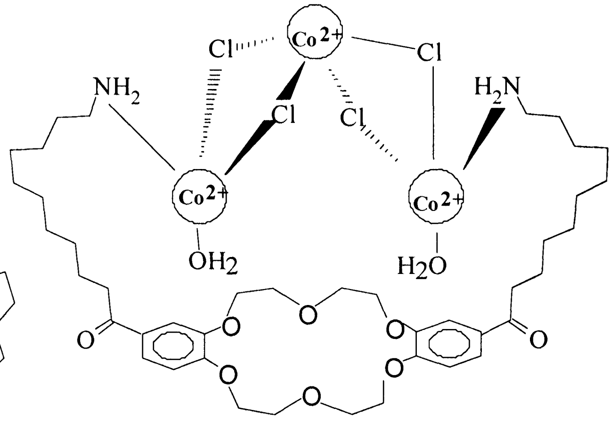

**7**

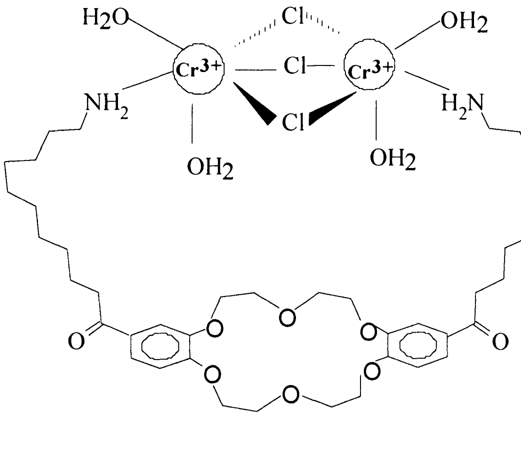

**8**

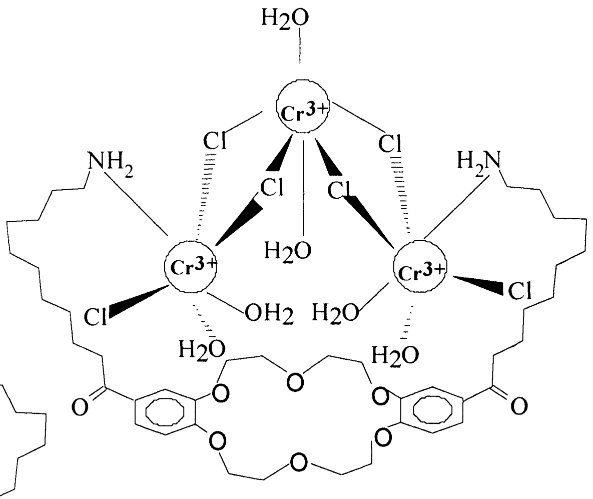

**9**

The diffuse reflectance spectra of the Ni(II) complexes $\mathbf{Ni_2Cl_2L(H_2O)_2}$ **4** and $\mathbf{Ni_4Cl_4L(H_2O)_4}$ **5** are also characteristic for a distorted (pseudo)-octahedral surrounding with the following transition bands: $\nu_1$: 11.89 x $10^3$ ($^3A_{2g}\rightarrow{}^3T_{2g}$), $\nu_2$: 14.59 x $10^3$ ($^3A_{1g}\rightarrow{}^3T_{1g}(F)$), 23.49 x $10^3$ $cm^{-1}$ ($^3A_{1g}\rightarrow{}^3T_{1g}(P)$) and $\nu_3$: 27.78 x $10^3$ $cm^{-1}$ (charge transfer band) for complex **4** (the $NiNO_3O'Cl$ as chromophore) and $\nu_1$: 12.92 x $10^3$ ($^3A_{2g}\rightarrow{}^3T_{2g}$), $\nu_2$: 14.79 x $10^3$ ($^3A_{1g}\rightarrow{}^3T_{1g}(F)$), $\nu_3$: 23.49 x $10^3$ $cm^{-1}$ ($^3A_{1g}\rightarrow{}^3T_{1g}(P)$) for complex **5**, respectively, (Table 2)(the $NiNO_2O'Cl$ and $NiCl_4O_2$ as chromophores).[14][15]

The complexes $\mathbf{CoCl_2L(H_2O)_2}$ **6** and $\mathbf{Co_3Cl_4L(H_2O)_4}$ **7** probably possess a distorted tetrahedral geometry of the metal ions, as confirmed by the presence of a large absorption $\nu_3$ band associated to the $^4A_2\rightarrow{}^4T_1(P)$ transition, which is resolved in two bands at 15.94 and 16.69 kK (complex **6**) and 14.35 and 15.92 kK (complex **7**).[16] The $\nu_2$ transition, overlapping with ligand transition bands, appeared at E < 10 kK being generally characteristic for tetrahedral Co(II) complexes (table 2).[16] Such spectra, characteristic for the cobalt(II) ion in a (pseudo)-tetrahedral geometry (the light-blue color of the compound is characteristic for the symmetric $C_{2v}$ surrounding), contain chromophores units of the type $CoN_2Cl_2$ for the complex **6** and $CoNOCl_2$ and $CoCl_4$ type for the complex **7**. [15][16]

The diffuse reflectance spectra of the Cr(III) derivatives $\mathbf{Cr_3Cl_4L(H_2O)_4}$ **8** and $\mathbf{Cr_3Cl_4L(H_2O)_{12}}$ **9** are also characteristic[14] for a distorted (pseudo)-octahedral surrounding with the following transition bands: $\nu_1$: 13.31 x $10^3$ ($^4A_{2g}\to{}^2T_{2g}$), $\nu_2$: 14.87 x $10^3$ ($^4A_{2g}\to{}^2E_g$), $\nu_3$: 23.24 x $10^3$ $cm^{-1}$($^4A_{2g}\to{}^4T_{2g}$), 27.28 x $10^3$ $cm^{-1}$($^4A_{2g}\to{}^4T_{1g}(F)$) and 37.37 ($^4A_{2g}\to{}^4T_{1g}(P)$) for complex **8** (the $CrNOCl_3$ chromophore) and $\nu_1$: 11.29 x $10^3$ ($^4A_{2g}\to{}^2T_{2g}$), $\nu_2$: 15.12 x $10^3$ ($^4A_{2g}\to{}^2E_g$), $\nu_3$: 22.49 x $10^3$ $cm^{-1}$($^4A_{2g}\to{}^4T_{2g}$), 27.28 x $10^3$ $cm^{-1}$($^4A_{2g}\to{}^4T_{1g}(F)$) and 36.76 ($^4A_{2g}\to{}^4T_{1g}(P)$) for complex **5** (Table 2) ($CrNO_2Cl_2$ and $CrO_2Cl_4$ chromophores).

*Biochemistry and biological activity of the new complexes*

Opportunistic fungal infections are an increasingly important cause of morbidity and mortality, with *Aspergillus* and *Candida* species being the most common such pathogens.[17, 18] Members of the genus *Aspergillus* are associated with an impressive spectrum of diseases in humans, ranging from benign colonization of the lung to severe pathologies such as invasive aspergillosis or allergic bronchopulmonary aspergillosis.[19] Although *A. fumigatus* has been identified as the most common etiological agent in the human diseases, being considered a pathogen and allergen at the same time,[19], [20] recent data showed the

Table 2: Electronic spectroscopic data for complexes **2-9**.

| Complex | Absorbtion band (kK) | Assigned transition |
|---|---|---|
| $\mathbf{Cu_2Cl_4L}$, **2** | 11.35 ($\nu_1$) | $d_{z^2}\to d_{x^2-y^2}$ |
| | 14.37($\nu_2$) | $d_{xy}\to d_{x^2-y^2}$ |
| | 17.50($\nu_3$) | $d_{xz}, d_{yz}\to d_{x^2-y^2}$ |
| $\mathbf{Cu_3Cl_4L(H_2O)_2}$ **3** | 11.89 ($\nu_1$) | $d_{z^2}\to d_{x^2-y^2}$ |
| | 14.59($\nu_2$) | $d_{xy}\to d_{x^2-y^2}$ |
| | 14.63 | $d_{xz}, d_{yz}\to d_{x^2-y^2}$ |
| | 18.549$\nu_3$) | charge transfer |
| $\mathbf{Ni_2Cl_2L(H_2O)_2}$, **4** | 11.89 ($\nu_1$) | $^3A_{2g}\to{}^3T_{2g}$ |
| | 14.59 ($\nu_2$) | $^3A_{1g}\to{}^3T_{1g}(F)$ |
| | 23.49 | $^3A_{1g}\to{}^3T_{1g}(P)$ |
| | 27.78 ($\nu_3$) | charge transfer |
| $\mathbf{Ni_4Cl_4L(H_2O)_4}$, **5** | 12.92 ($\nu_1$) | $^3A_{2g}\to{}^3T_{2g}$ |
| | 14.79 ($\nu_2$) | $^3A_{1g}\to{}^3T_{1g}(F)$ |
| | 23.49 | $^3A_{1g}\to{}^3T_{1g}(P)$ |
| $\mathbf{CoCl_2L(H_2O)_2}$, **6** | E<10 ($\nu_2$) | $^4A_2\to{}^4T_1(F)$ |
| | 15.94 ($\nu_3$) | $^4A_2\to{}^4T_1(P)$ |
| | 16.69 | |
| $\mathbf{Co_3Cl_4L(H_2O)_2}$ **7** | E<10 ($\nu_2$) | $^4A_2\to{}^4T_1(F)$ |
| | 14.35 ($\nu_3$) | $^4A_2\to{}^4T_1(P)$ |
| | 15.92 | |
| $\mathbf{Cr_3Cl_4L(H_2O)_4}$ **8** | 13.31 ($\nu_1$) | $^4A_{2g}\to{}^2T_{2g}$ |
| | 14.87 ($\nu_2$) | $^4A_{2g}\to{}^2E_g$ |
| | 23.24 | $^4A_{2g}\to{}^4T_{2g}$ |
| | 27.28 | $^4A_{2g}\to{}^4T_{1g}(F)$ |
| | 37.37 ($\nu_3$) | $^4A_{2g}\to{}^4T_{1g}(P)$ |
| $\mathbf{Cr_3Cl_4L(H_2O)_1}$ **9** | 11.29 ($\nu_1$) | $^4A_{2g}\to{}^2T_{2g}$ |
| | 15.12 ($\nu_2$) | $^4A_{2g}\to{}^2E_g$ |
| | 22.49 | $^4A_{2g}\to{}^4T_{2g}$ |
| | 27.28 | $^4A_{2g}\to{}^4T_{1g}(F)$ |
| | 36.76 ($\nu_3$) | $^4A_{2g}\to{}^4T_{1g}(P)$ |

apparently benign *A. niger* and *flavus* to be involved in life-threatening conditions such as fungal endocarditis[21] as well as endogenous endophthalmitis, leading in many cases to an irreversible loss of visual outcome.[22] Moreover, these and other fungi developed resistance to many of the clinically used drugs, such as ketoconazole **10** or itraconazole **11**, so that novel pharmacological agents of this type are permanently needed.[19-22]

The mechanism of action of many fungistatic drugs, such as the widely clinically used azoles mentioned above, **10** and **11**, [23-26] consists in inhibition of a metallo-enzyme, sterol 14-α-demethylase (CYP51A1), which is a microsomal cytochrome P-450 dependent protein belonging to a gene superfamily involved in sterol biosynthesis in fungi, plants and animals.[27-29] CYP51A1 has been shown to catalyze the conversion of lanosterol **12** to the 14-desmethylated derivative, ergosterol **13**, through a complicated oxidative sequence involving 4,4-dimethylcholesta-8,24-dienol and 4,4-dimethyl-cholesta-8,14,24-trienol, as well as the $CH_2OH$, CHO and COOH derivatives corresponding to the 14-methyl carbon atom of lanosterol, followed by

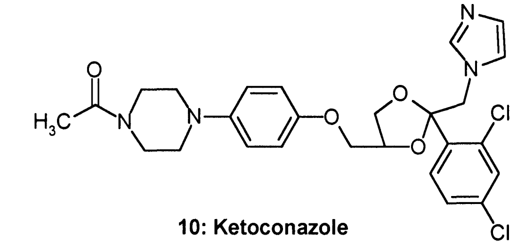

**10: Ketoconazole**

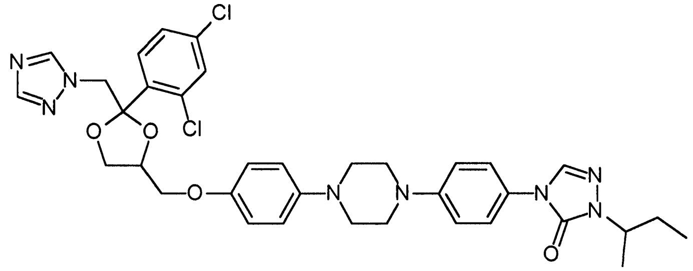

**11: Itraconazole**

decarboxylation of the latter compound and release of formic acid.[30-32] Inhibition of CYP51A1 by azole antifungals causes thus depletion of ergosterol and accumulation of 14-methylsterols in the membrane of fungal cells, disturbing the membrane function and causing the death of these organisms.[18,24-30]

Taking into account our interest for the design and preparation of novel biologically active coordination compounds, we have recently reported several classes of antifungals such as aminoglutethimide derivatives[33], phenoxathiins,[34] phenoxathiin-10,10-dioxides,[35] phenazines,[36] as well as some of their metal complexes.[33,37] Such compounds generally showed a large variety of interesting biological activities, possessing among others antifungal activity against several fungi such as *Aspergilli* and *Candida*.[33-37] Some of these compounds were shown to interact with the ergosterol synthesis pathway in the sensitive fungi *A.*

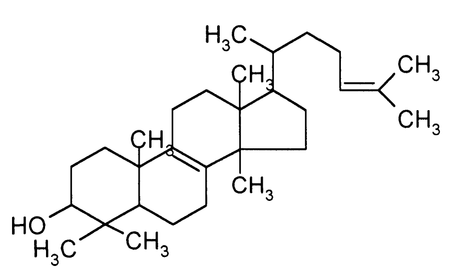

**12: lanosterol**

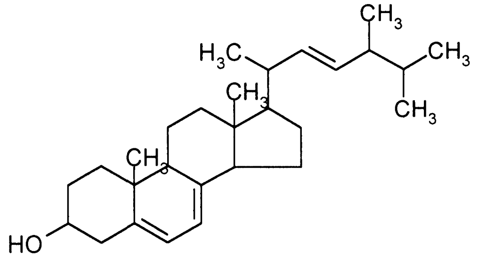

**13: ergosterol**

*niger*,[34,35,37] possessing thus a similar mechanism of action with the azole antifungals. It appeared thus of great interest to test whether the coordination compounds of the crown ether derivative **1**, of the type **2-8**, possess antifungal activity. The rationale of looking for the antifungal activity of compounds of the type reported in the present paper is the following one: substrates of CYP51A1 and related enzymes are generally bulky molecules (sterols possessing several bulky side chains) which must be oxidized at a specific methyl group. Thus, their interaction with the active site Fe(III) ion of the prosthetic group of cytochrome P450 is governed by a multitude of both hydrophobic as well as polar interactions.[17] The same mechanism is valid for the interaction between CYP51A1 and its inhibitors (such as the azole antifungals). Moreover, many antifungal compounds (see for instance the structure of itraconazole) possess themselves a bulky and relatively rigid structure, together with one or more highly polar moieties which are important for their interaction with the prosthetic group as well as with other active site residues in its vicinity. Thus, our hypothesis was that compounds of the type **2-8** prepared by us, might possess such structural elements, which would confer them the desired biological activity, i.e., antifungal action. Mention should be made that the crown-ether **1** possesses modest antifungal properties, which were discovered casually by us during a normal screening program for detecting new lead molecules against the three fungi used in the experiments, i.e., *A. niger*, *A. flavus* and *C. albicans* (Table 3).

Table 3: Antifungal activity of compounds **1-11** against several organisms. MIC's have been determined as described in ref. [38]

| Compound | MIC (μg/mL) *A. flavus* C1150 | *A. niger* C418 | *Candida albicans* C316 |
|---|---|---|---|
| **1** | 75 | 70 | 35 |
| **2** | 2 | 1 | 9 |
| **3** | 3 | 2 | 10 |
| **4** | 7 | 8 | 10 |
| **5** | 12 | 9 | 14 |
| **6** | 6 | 5 | 9 |
| **7** | 9 | 6 | 10 |
| **8** | 8 | 5 | 9 |
| **9** | 10 | 7 | 13 |
| **Ketoconazole 10** | 1.2 | 1.8 | 0.06 |
| **Itraconazole 11** | 0.9 | 0.2 | 0.02 |

Table 4: Levels of ergosterol in *A. niger* cultures after treatment with different concentrations of the azole CYP51A1 inhibitor itraconazole **11** and compound **2**.

| Inhibitor | Concentration (μg/mL) | % Ergosterol* |
|---|---|---|
| Itraconazole **11** | 0.01 | 78 ± 5 |
| Itraconazole **11** | 0.05 | 41 ± 7 |
| Itraconazole **11** | 0.10 | 11 ± 4 |
| **2** | 0.01 | 92 ± 3 |
| **2** | 0.10 | 63 ± 5 |
| **2** | 0.30 | 29 ± 6 |
| **2** | 0.85 | 8 ± 2 |

*Mean ± standard deviation (n = 3); The amount of ergosterol present in the same amount of wet cells from the culture grown in the absence of inhibitor is taken as 100%.

From data of Table 3 it can be seen that the ligand **1** possesses modest but net antifungal activity against the three fungi species used in our experiments, whereas the metal complexes of **1** act as much more effective antifungals than the parent ligand, with potencies sometimes comparable to ketoconazole against the *Aspergilli*, but being much less efficient against *Candida* as compared to the azole antifungals. The metal ion present in the coordination compound is an important factor for the biological activity, with Cu(II) derivatives more active than Co(II) complexes, which in turn were more active than the Ni(II) and Cr(III) derivatives. It is also interesting to note that the di- or mononuclear complexes were more active than the corresponding trinuclear derivatives (compare **2** and **3**, or **4** and **5**, **6** and **7**, **8** and **9**). The species most susceptible to inhibition by these antifungals was *A. niger*, followed by *A. flavus*, whereas *C. albicans* was the most resistant. In fact, against the first fungus, the Cu(II) derivative **2** for instance is more effective than ketoconazole, a widely used drug, being only slightly less effective than itraconazole, one of the most potent antifungals known up to now. [17,23,24]

In order to test the hypothesis that the compounds reported here act as ergosterol biosynthesis inhibitors, similarly to the azole antifungals, the amounts of ergosterol present in *A. niger* cultures after treatment with different concentrations of new inhibitors (itraconazole **11**, a potent CYP51A1 inhibitor[17,23,24] has also been included in the study as standard) have been determined by means of a HPLC method (Table 4).[39] These data show that at low concentrations of inhibitor, around 80-96 % of ergosterol (as compared to the amount of sterol formed in cultures in which inhibitors have not been added, and which was considered 100%) is still synthesized. By increasing the concentrations of inhibitors used in the experiments, the amount of synthesized ergosterol decreased dose-dependently. A similar effect has been observed for the well-known CYP51A1 inhibitor itraconazole **11** as well as for the new antifungal compound **2** synthesized in the present study (the most active inhibitor in the series). These data allow us to propose a similar mechanism of action for the two classes of antifungal compounds, i.e., the inhibition of lanosterol-14-α-demethylase, although it is not improbable that our compounds might interfere with (an)other enzyme(s) involved in the ergosterol biosynthetic pathway. Thus, we presume that our original hypothesis that bulky coordination compounds of the type described in the present paper might interfere with the prostethic group of CYP51A1, is correct. Presently it is difficult to make a hypothesis regarding the precise mode of interaction between the inhibitor and the CYP51A1 active site, but we consider the carbonyl oxygen or the amino group of derivatives **1-9** as the most probable candidates for binding to the Fe(III) ion of this metallo-enzyme. In this way one could also explain why the metal complexes are much more active as compared to the parent ligand **1**: thus, **1** possesses a much more flexible molecule as compared to the metal complexes **2-9**, in which the presence of the metal ion(s) somehow produces much more rigid structures. Such rigid structures probably fit better and bind tighter to the active site of CYP51A1 as compared to **1**, and in consequence the metal complexes act as better antifungals.

**Materials and Methods**

IR spectra: CsBr pellets, 400-4000 $cm^{-1}$ Nicolet 2DXFTIR instrument. -Electronic spectra: MgO as reference, 300-1100 nm, diffuse reflectance - Elemental analysis: Carlo Erba Instrument CHNS Elemental Analyser, Model 1106. Reverse-phase HPLC: Beckman instrument, on a μ-Bondapak-C18 column, with acetonitrile as eluting solvent. 2,3,11,12-bis[4-(10-aminodecylcarbonyl)]benzo-18-crown-6, **1** was prepared by the method previously reported by us [8]; metal salts and solvents from Fluka or E. Merck were used without purification. Ketoconazole and itraconazole were from Janssen, whereas ergosterol and lanosterol used as standards in the HPLC measurements were from Sigma.

*Synthesis of complexes containing 2,3,11,12-bis[4-(10-aminodecylcarbonyl)]benzo-18-crown-6, 1 as macrocyclic ligand* : 4 $10^{-4}$ mol (0.29 g) of **1** (**L**) dissolved in 25 ml $CHCl_3$ and and 25 ml of methanolic solutions containing metal salts ($MCl_x$ y $H_2O$: M=Cu(II), y=2; Ni(II),, y=6; Co(II), y=3; Cr(III), y=6) were mixed with stirring, working at molar ratio L : M of 1:1 (4 $10^{-4}$ mol : 6.84 $10^{-2}$ g $CuCl_2$ $2H_2O$, 9.48 $10^{-2}$ g $NiCl_2$ $6H_2O$, 7.36 $10^{-2}$ g $CoCl_2$ $3H_2O$, 1.06 $10^{-1}$ g $CrCl_3$ $6H_2O$) and 1:2 (8 $10^{-4}$ mol: 1.36 $10^{-1}$ g $CuCl_2$ $2H_2O$, 1.89 $10^{-1}$ g $NiCl_2$ $6H_2O$, 1.47 $10^{-1}$ g $CoCl_2$ $3H_2O$, 2.13 $10^{-1}$ g $CrCl_3$ $6H_2O$), respectively. The resulting mixtures were refluxed for 60 min. on a water bath. The complexes precipitated from these solutions were filtered, washed with methanol and diethyl ether and dried in vacuum The following coordination compounds were synthesized and characterized: $Cu_2Cl_4L$ (yellow-green) **2**, $Cu_3Cl_4L(H_2O)_2$, (brown), **3**, $Ni_2Cl_2L(H_2O)_2$ , (yellow) **4**, $Ni_4Cl_4L(H_2O)_4$ (yellow) **5**, $CoCl_2L(H_2O)_2$ (light blue) **6**, $Co_3Cl_4L(H_2O)_4$ (light green-blue) **7**, $Cr_3Cl_4L(H_2O)_4$ (brown-green), **8**, $Cr_3Cl_4L(H_2O)_{12}$ (brown-green), **9**. The yields were in the range of 45-82% : 0.26 g of **2** (65%), 0.27 g of **3** (62%), 0.17 g of **4** (45%), 0.24 g of **5** (52%), 0.29 g of **6** (82%), 0.24 g of **7** (55%), 0.25 g of **8** (58%), 0.25 g of **9** (50%)).

**$Cu_2Cl_4L$ 2**: IR (CsBr) $cm^{-1}$: 951, 1055, 1133, 1264, 1427, 1455, 1514, 1595, 1673, 2853, 2926, 3205, 3448. UV-VIS (MgO), $cm^{-1}$x $10^{-3}$: 11.35, 14.37, 17.50. Analysis: $Cu_2Cl_4L$ (996) found Cu 12.42, C 51.28, H 6.86, N 2.47, Cl 14.00; requires Cu 12.76, C 50.65, H 6.68, N 2.81, Cl 14.24.

**$Cu_3Cl_4L(H_2O)_2$ 3**: IR (CsBr) $cm^{-1}$: 950, 1058, 1134, 1264, 1429, 1460, 1514, 1595, 1674, 2854, 2930, 3205, 3460. UV-VIS (MgO), $cm^{-1}$x $10^{-3}$: 11.89, 14.59, 14.63, 18.49. Analysis: $Cu_3Cl_4L(H_2O)_2$ (1096) found Cu 17.58, C 46.48, H 6.57, N 2.47, Cl 14.28; requires Cu 17.69, C 46.82, H 6.36, N 2.60, Cl 14.16.

**$Ni_2Cl_2L(H_2O)_2$ 4**: IR (CsBr) $cm^{-1}$ : 876, 953, 1056, 1131, 1148, 1264, 1337, 1428, 1456, 1514, 1596, 1673, 2854, 2927, 3198, 3405. UV-VIS (MgO), $cm^{-1}$x $10^{-3}$: 11.89, 14.59, 23.49, 27.78. Analysis: $Ni_2Cl_2L(H_2O)_2$ (949) found Cu 12.35, C 65.53, H 7.42, N 2.94, Cl 9.45; requires Ni 12.36, C 65.75, H 7.36, N 2.60, Cl 14.16.

**$Ni_4Cl_4L(H_2O)_4$ 5**: IR (CsBr) $cm^{-1}$ : 875, 953, 1056, 1131, 1148, 1264, 1337, 1428, 1456, 1514, 1596, 1673, 2853, 2927, 3368. UV-VIS (MgO), $cm^{-1}$x $10^{-3}$: 12.92, 14.79, 23.49. Analysis: $Ni_4Cl_4L(H_2O)_4$ (1172) found Ni 19.54, C 43.05, H 6.78, N 2.64, Cl 12.32; requires Ni 19.97, C 42.91, H 6.34, N 2.38, Cl 12.06.

**$CoCl_2L(H_2O)_2$ 6**: IR (CsBr) $cm^{-1}$ : 876, 953, 1056, 1132, 1148, 1266, 1337, 1359, 1428, 1456, 1514, 1596, 1674, 2854, 2926, 3437. UV-VIS (MgO), $cm^{-1}$x $10^{-3}$: <10.0, 15.94, 16.69. Analysis: $CoCl_2L(H_2O)_2$ (892) found Co 6.34, C 56.89, H 7.46, N 4.62, Cl 8.56; requires Co 6.60, C 56.6, H 7.83, N 4.14, Cl 8.14.

**$Co_3Cl_4L(H_2O)_4$ 7**: IR (CsBr) $cm^{-1}$ : 842, 952, 1055, 1125, 1149, 1264, 1335, 1360, 1426, 1457, 1513, 1596, 1673, 2854, 2927, 3170, 3554. UV-VIS (MgO), $cm^{-1}$x $10^{-3}$: <10.0, 14.35, 15.92. Analysis: $Co_3Cl_4L(H_2O)_4$ (1117) found Co 11.28, C 49.1, H 7.20, N 0.80, Cl 14.12; requires Co 11.52, C 49.3, H 6.90, N 0.81, Cl 13.87.

**$Cr_3Cl_4L(H_2O)_4$ 8**: IR (CsBr) $cm^{-1}$: 803, 951, 1056, 1129, 1148, 1149, 1275, 1339, 1429, 1464, 1517, 1596, 1676, 2854, 2927, 3173. UV-VIS (MgO), $cm^{-1}$x $10^{-3}$: 13.31, 14.87, 23.24, 27.28, 36.76. Analysis: $Cr_3Cl_4L(H_2O)_4$ (1096) found Cr 10.64, C 49.56, H 7.85, N 2.60, Cl 10.05; requires Cr 10.30, C 49.98, H 7.39, N 2.78, Cl 10.54.

**$Cr_3Cl_4L(H_2O)_{12}$ 9**: IR (CsBr) $cm^{-1}$: 786, 875, 952, 1056, 1130, 1149, 1264, 1340, 1429, 1464, 1516, 1596, 1673, 2853, 2927, 3074, 3132, 3156, 3404. UV-VIS (MgO), $cm^{-1}$x $10^{-3}$: 11.29, 15.12, 22.49, 27.28, 36.76. Analysis: $Cr_3Cl_4L(H_2O)_{12}$ (1240) found Cr 12.03, C 38.30, H 7.10, N 1.85, Cl 15.98; requires Cr 11.89, C 38.45, H 6.91, N 2.14, Cl 16.21.

*Assay of fungistatic activity of compounds* **1-11**

Fungistatic activity was determined by a modification of the growth method recently reported by us,[33-37] utilizing two *Aspergillus* and one *Candida spp.* Minimum inhibitory concentrations (MICs) have been determined by the agar dilution method with Iso-Sensitest agar as described by Kinsman et al.[38] The fungi were cultivated in agar plates at 37°C for 5 days, in the nutrient broth (NB, Diagnostic Pasteur), in the absence and in the presence of 100 - 0.01 µg/mL of compounds **1-11**. Stock solutions of inhibitors were obtained in DMSO (100 mg/mL) and dilutions up to 0.01 µg/mL were done with distilled deionized water. The minimum concentration at which no growth was observed was taken as MIC value (µg/mL), and represents the mean of at least two determinations. Standard deviations were generally around 2 – 3 % (data not shown).

*Assay of sterols present in the fungi cultures*

A reverse-phase HPLC method adapted from the literature,[39] has been used to determine the amount of sterols (ergosterol **12** and lanosterol **13**) present in the fungi cultures. HPLC was performed with a Beckman instrument, using a Rheodyne pump and column (reverse phase µ-Bondapack-C18). The fungi have been cultivated as mentioned above for 5 days, with or without inhibitors added in the nutrient broth. Culture media were suspended in a small volume of MOPS buffer (pH 7.4) and the cells centrifuged at 20,000g for 30 min. Cells were weighed (wet paste) and broken by sonication. Sterols present in the homogenate were then extracted in chloroform, the solvent has been evaporated to a small volume and the extracts applied on a µ-Bondapak-C18 column, with acetonitrile as eluting solvent. Authentic ergosterol and lanosterol (from Sigma) were used as standards. The flow rate was of 3 mL/min. The retention times were: 8.87 min for ergosterol; and 7.62 min for lanosterol, respectively. Blank assays were done for cultures which were not treated with inhibitors in order to assess the normal levels of sterols present. The amount of ergosterol present in the same amount of wet cells from the culture grown in the absence of inhibitor was taken as 100%.[40]

**References**

[1] C. J. Pedersen, *J. Am. Chem. Soc.* **1967**, *89*, 2495-2496.
[2] C. J. Pedersen, *J. Am. Chem. Soc.* **1967**, *89*, 7017-7036.
[3] G.W. Gokel, E. Abel in *Comprehensive Supramolecular Chemistry*, *Vol* 1 (Eds.: J.L. Atwood, J. E. D. Davies, D. D. MacNicol, F. Vögtle, K. S. Suslick), Pergamon, Oxford, **1996**, pp.511-534.
[4] J.M. Lehn, *La Chimie Supramoléculaire. Concepts et Perspectives* (in French), De Boeck & Larcier, Paris, Bruxelles, **1997**, pp. 17-24.
[5] R.D. Rogers, C.B. Bauer in *Comprehensive Supramolecular Chemistry*, *Vol* 1 (Eds.: J.L. Atwood, J. E. D. Davies, D. D. MacNicol, F. Vögtle, K. S. Suslick), Pergamon, Oxford, **1996**, pp. 315-355.
[6] P.C. Junk, S.M. Lynch, B.J. McCool, *Supramolecular Chemistry*, **1998**, *9*, 151-158.
[7] C.T. Supuran, G. Botez, M. Barboiu, O. Maior, A.T. Balaban, *Rev. Roum Chim.*, **1992**, *37*, 141-148.
[8] M. Barboiu, C.T. Supuran, C.Luca, G. Popescu, C. Barboiu, *Liebiegs. Ann.*, **1996**, 959-963.
[9] M. Barboiu, C. Luca, C.T. Supuran, A. Scozzafava, F. Briganti, N. Hovnanian, L. Cot, *Liebiegs. Ann./Recueil*, **1997**, *9*, 1853-1859.
[10] M. Barboiu, Ph.D. Disertation, University II of Montpellier, France, **1998**.
[11] M. Barboiu, C. Luca, C. Guizard, N. Hovnanian, L. Cot, G. Popescu, , *J. Membrane Sci*, **1997**, *129*, 197-207.
[12] M. Barboiu, N. Hovnanian, C. Luca, G. Popescu, L. Cot, *Eur. J. Org. Chem.*, **1998**, 1705-1708.
[13] R. Nakamoto, "Infrared and Raman Spectra of Inorganic Coordination Compound", Wiley, New York, **1986**.
[14] A.B.P. Lever, "Inorganic Electronic Spectroscopy" Elsevier Publishing Co., Amsterdam, **1984**, pp. 178-196.
[15] L. Sacconi, F.Maini, A.Becini, "Nickel", in Comprehensive Coordination Chemistry", G. Wilkinson, R. Gillard, J. McCleverty Eds., Pergamon Press, Oxford, **1987**, Vol 5, pp. 1-347.
[16] L. Banci, A. Becini, C.Benelli, D. Gatteschi, C. Zanchini, *Struct. Bonding*, **1982**, *52*, 37-79.
[17] D. Berg, M. Plempel, "Fungal enzyme targets", In "Design of Enzyme Inhibitors as Drugs", M. Sandler, H.J. Smith Eds., Oxford Univ. Press, Vol. 2, pp. 364-377, 1994.
[18] a) M. Mallie, J.M. Bastide, *Drugs Exp. Clin. Res.*, **1996**, *22*, 301-307; b) Y. Yoshida, *Curr. Top. Med. Mycol.*, **1988**, *2*, 388-418.
[19] R. Crameri, *Int. Arch. Allergy Immunol.*, **1998**, *115*, 99-114.

[20] D.W. Denning, S.A. Radford, K.L. Oakley, L. Hall, E.M. Johnson, D.W. Warnock, *J. Antimicrob. Chemother.*, **1997**, *40*, 401-414.
[21] Z.U. Khan, S.C. Sanyal, E. Mokaddas, I. Vislocky, J.T. Anim, A.L. Salama, H. Shuhaiber, *Mycoses* **1997**, *40*, 213-217.
[22] P.D. Weishaar, H.W. Flynn, T.G. Murray, J.L. Davis, C.C. Barr, J.G. Gross, C.E. Mein, W.C. McLean, J.H. Killian, *Ophthalmology* **1998**, *105*, 57-65.
[23] H. Vanden Bossche, *Drug Dev. Res.*, **1986**, *8*, 287-298.
[24] a) P. Marichal, H. Vanden Bossche, *Acta Biochim. Pol.*, **1995**, *42*, 509-516; b) F.C. Odds, *J Antimicrob. Chemother.*, **1993**, *31*, 463-471.
[25] T. Joseph-Thorne, D.W. Hollomon, *FEMS Microbiol. Lett.*, **1997**, *149*, 141-149.
[26] T.C. White, *Antimicrob. Agents Chemother.*, **1997**, *41*, 1488-1494.
[27] L.M. Koymans, H. Moereels, H. Vanden Bossche, *J. Steroid. Biochem. Mol. Biol.*, **1995**, *53*, 191-197.
[28] D. Rozman, M. Stromstedt, L.C. Tsui, S.W. Scherer, M.R. Waterman, *Genomics,* **1996**, *15*, 371-381.
[29] Y. Aoyama, M. Noshiro, O. Gotoh, S. Imaoka, Y. Funae, N. Kurosawa, T. Horiuchi, Y. Yoshida *J. Biochem.*, **1996**, *119*, 926-933.
[30] C.A. Hitchcock, S.B. Brown, E.G. Evans, D.J. Adams, *Biochem. J.*, **1989**, *260*, 549-556.
[31] R.T. Fischer, S.H. Stam, P.R. Johnson, S.S. Ko, R.L. Magolda, J.L. Gaylor, J.M. Trzaskos, *J. Lipid Res.*, **1989**, *30*, 1621-1632.
[32] S. Bak, R.A. Kahn, C.E. Olsen, B.A. Halkier, *Plant. J.*, **1997**, *11*, 191-201.
[33] F. Briganti, A. Scozzafava, C.T. Supuran, *Eur. J. Med. Chem.*, **1997**, *32*, 901-910.
[34] C.T. Supuran, A. Scozzafava, F. Briganti, G. Loloiu, O. Maior, *J. Enzyme Inhib.*, **1998**, *13*, 291-310.
[35] A. Nicolae, T. Cristea, C.T. Supuran, *Rev. Roum. Chim.*, **1997**, *42*, 301-306.
[36] a) C.T. Supuran, A. Scozzafava, F. Briganti, G. Loloiu, O. Maior, *Eur. J. Med. Chem.*, **1998**, *33*, in press; b) A. Scozzafava, A. Nicolae, O. Maior,F. Briganti, C.T. Supuran, *J. Enzyme Inhib.*, **1998**, *14*, 49-68.
[37] C. Guran, M. Barboiu, P. Diaconescu, V. Iluc, M. Bojin, A. Scozzafava, C.T. Supuran, *Metal Based Drugs*, **1998**, *5*, 287-294.
[38] O.S. Kinsman, D.G. Livermore, C. Smith, *Antimicrob. Agents Chemother.*, **1993**, *37*, 1242-1246.
[39] a) E. Hansbury, T.J. Scallen, *J. Lipid Res.*, **1978**, *19*, 742-746; b) B. Ruan, N. Gerst, G.T. Emmons, J. Shey, G.J. Schroepfer, *J. Lipid Res.*, **1997**, *38*, 2615-2626.
[40] a) Y. Aoyama, Y. Yoshida, Y. Sonoda, Y. Sato, *Biochim. Biophys. Acta,* **1991**, *1081*, 262-266; b) Y. Aoyama, Y. Yoshida, Y. Sonoda, Y. Sato, *Biochim. Biophys. Acta,* **1992**, *1122*, 251-255; c) Y. Aoyama, Y. Yoshida, *Biochem. Biophys. Res. Commun.*, **1992**, *183*, 1266-1272.

**Received: January 27, 1999 - Accepted: February 11, 1999 - Received in revised camera-ready format: March 19, 1999**

